# Supplementary material for: Are carbon emissions trading and green financial instruments synergistic? -Comprehensive quantitative research based on content analysis
Source: PLoS One. 2024 Mar 7;19(3):e0298601. doi: 10.1371/journal.pone.0298601 (PMC10919601; doi:10.1371/journal.pone.0298601)
Supplement: S1 Data — (ZIP) [file pone.0298601.s001.zip › Data/地级市Z_F工作报告.zip!/║╙▒▒/╩»╝╥╫»╩╨/└┤╫╘╣1⁄2╓┌║┼í╛┬φ┐╦╩2╛▌═°í┐_2015.docx]

各位代表：

现在，我代表市人民政府向大会作工作报告，请予审议，并请市政协各位委员和列席会议的同志提

出意见。

一、2014年工作回顾

刚刚过去的2014年是极不平凡的一年，面对宏观经济下行压力不断加大和环境约束日益趋紧的严峻形势，全市人民在省委、省政府和中共石家庄市委的正确领导下，全力以赴稳增长、调结构、促改革、治污染、惠民生，实现了经济社会稳步健康发展，较好地完成了市十三届人大二次会议确定的目标任务。

经济运行稳中向好。坚持把稳增长摆在更加重要的位置，突出投资拉动，强化要素保障，全市经济平稳健康运行，主要经济指标在全省保持领先。预计地区生产总值完成5100亿元，增长8%，高于全省1.5个百分点。全部财政收入完成681.3亿元，可比增长10%；一般公共预算收入完成343.5亿元，可比增长13%，总量均居全省第一。固定资产投资完成5076亿元，增长16.2%。社会消费品零售总额完成2424亿元，增长12.5%。外贸进出口总值完成147亿美元，增长5%。民营经济增加值完成3400亿元，增长8.5%，全省综合排名位居第一。城乡居民人均可支配收入分别达到27422元、11123元，增长8.5%和10.5%。

结构调整取得明显进展。牢牢抓住经济结构调整这个战略重点，有保有压、有扶有控，实现了经济发展与结构优化良性互动。项目建设势头强劲。全市亿元以上在建项目达到889个，光谷一期等145个攻坚项目进展顺利，完成年度投资计划的120%。中博新能源汽车、三元工业园等51个新开工项目加快建设，石炼化800万吨油品升级改造、常山生化肝素钠、君乐宝婴幼儿配方乳粉等48个重点项目竣工投产。25个省级以上开发区主营业务收入完成6152.8亿元，增长19.7%。工业转型升级步伐加快。坚持“有中生新”，改造提升传统产业，集中实施技改项目1600项，完成技改投资1536亿元，增长21%；工业企业全面开展对标行动，创建省级对标示范企业26家、行业标杆543项；新增入统工业企业200家，规模以上工业增加值完成2072亿元、利润达到746亿元，分别增长8%和11%。坚持“无中生有”，培育壮大新兴产业，生物医药产业列入国家高端生物医药区域集聚发展试点，中航通用飞机、格力电器等143项战略性新兴产业项目建设提速，新增高新技术企业76家，高新技术产业实现增加值310亿元，增长13%。服务业快速发展。完成增加值2169亿元，对经济增长的贡献率达到53%。成功列入国家电商与物流快递业协调发展试点城市和全国电子商务进农村综合示范项目计划，商务云数据中心建成投用，电商交易额达到2400亿元，增长25%。三大省级物流园区入驻商户1.3万家，年交易额突破400亿元。国际贸易城、华润万象城等121个商贸重点项目扎实推进。成功举办首届石家庄旅游交易会，旅游业总收入达到406亿元。金融保险、文化创意、养老服务等产业健康发展，北京银行石家庄分行正式营业。农业生产稳定增长。国家现代农业示范区申报成功。粮食生产获得丰收，连续三次获评全国粮食生产先进市。肉、蛋、奶和蔬菜、果品产量全省领先。积极培育新型农业经营主体，家庭农场由54家增加到770家，农民合作社达到1.2万家。实施农业产业化项目184个，新增省、市重点龙头企业45家，平山葫芦峪等农业综合开发项目取得成效，农业产业化经营率达到61.6%。

改革开放扎实推进。重点领域改革全面深化，市场活力和内生动力明显增强。政府职能转变步伐加快。衔接落实省政府取消下放的审批事项97项，向县（市）区下放审批事项45项，市级保留的113项审批项目全部向社会公开。食药监和工商、质监行政体制改革基本完成。市本级44个行政机关标准化建设全面完成。经济领域改革不断深化。全面落实商事制度改革措施，新增各类市场主体10.2万户，增长44%；新登记注册资本1538.6亿元，增长165.2%。完成市机械供销公司等8家国有企业改制，21家监管企业实现营业收入293亿元、利润6.3亿元，双超年度计划。15个试点村完成土地承包经营权确权颁证，农村土地承包经营权流转达到159万亩，占家庭承包耕地的24.5%。省会的开放度进一步提升。石家庄综合保税区获国务院批准；成功组织石洽会等系列招商引资活动，全年实际利用外资10.2亿美元，引进市外资金1353亿元；新增多层次资本市场挂牌上市企业30家、境外投资企业27家，超千万美元进出口企业达到92家。创新发展能力持续提高。科技大市场建成投用，与中国技术交易所等15家技术转移机构开展合作，中德医疗器械联合实验室正式揭牌，中欧联合实验室获得中国、美国、加拿大检测体系认证。科技中心等省级以上孵化器达到9家，孵化器总面积达到100万平方米，入驻企业667家。协同创新发展迈出坚实步伐。明确了石家庄在京津冀协同发展中打造现代商贸物流基地的功能定位。由我市发起和组织的京津冀产学研联盟正式成立，北大、清华、中科院等242家知名院校、科研机构和企业加入。成功签署与中关村共建集成电路产业基地协议。与央企合作不断深化，首安工业自动灭火系统等97个项目顺利推进。

新型城镇化进程加快。强力推进大省省会建设，全市城镇化率达到56.3%。省会发展空间实现大拓展。顺利完成行政区划调整，城区面积由469平方公里扩展到2206平方公里。交通枢纽建设步伐加快，石济客专正式启动，京港澳高速改扩建竣工通车，京昆石太高速、南绕城高速顺利推进；307国道大修完工，308国道改造加快实施，新赵公路全线贯通，新建干线公路110公里；太行大街、新城大道建成通车，把“一河两岸”连为一体。城市载体功能不断提升。正定国际机场第二航站楼、新客站东广场建成投用；轨道交通1、3号线一期工程55个工点全部开工，14个地铁站主体封顶；新胜利大街、方北路和裕翔街等城市路网建设改造整体加快。竣工热源项目80个，新增供热面积963万平方米。实施石津干渠沿线雨污导排工程，完成主城区4条路段雨污分流改造。南水北调一期工程全线通水。对槐安路、裕华路、体育大街等主路主街进行了景观整治，改善提升老旧小区279个、小街巷100条，新建提升绿地710公顷。深入开展房地产专项整治，有效规范了房地产市场秩序。新区建设步伐加快。河北奥体中心、石家庄报业传媒大厦等24个功能性项目建设全面展开，湖南大道、天祥街等6条道路建成通车。正定古城墙保护、周汉河综合整治、月城、瓮城修复等十大工程60个项目顺利实施。县城建设力度加大。13个县（市）全部完成城乡总体规划编制。改造提升迎宾路101公里、标志性街道46公里，拆除违章建筑420万平方米，县城机械化清扫率达到50%。新增公共绿地741.6公顷，鹿泉、高邑通过国家级园林县城初评，井陉通过省级园林县城复查，新乐成功创建省级园林县城，高邑、元氏获省人居环境进步奖。368个省级重点村面貌改造提升任务全面完成，全国农村改厕工作现场会在正定召开。

生态环境持续改善。以壮士断腕的决心和勇气，全力实施环境治理攻坚战。大气质量明显好转。市财政投入资金15.8亿元，综合实施压煤、抑尘、控车、迁企等六大举措，全年空气质量优良天数达114天，比上年增加71天，优良率提高19.5个百分点，重度以上污染天数比上年减少51天，下降14个百分点，六项主要污染物平均浓度全面下降，其中PM2.5、PM10分别下降19.5%、32.5%，二氧化硫、一氧化碳、二氧化氮、臭氧分别下降41%、24.9%、22.1%、15.6%。率先在全省完成污染物来源解析。压减燃煤700万吨、钢铁产能112万吨、水泥产能1850万吨，提前3年完成水泥过剩产能压减任务。实施减排项目205项，全市17家热电企业实施除尘改造，市内所有工地开展绿色施工。淘汰黄标车8.07万辆。提前一年完成“十二五”节能减排任务。华旭药业、永通化工等4家企业完成搬迁，石钢搬迁至井陉矿区工作正式启动。生态修复力度加大。完成水污染治理项目469项，滹沱河整治二期工程进展顺利，汪洋沟综合整治工程基本完工，洨河出市断面水质稳定达到省考核要求；建立黄壁庄水库上游水源联动预警机制，拆除岗南水库周边违章建筑2万平方米，集中式饮用水源地水质稳定达标。大力实施环省会经济林建设等重大绿化工程，完成造林62万亩，超出目标12万亩，森林覆盖率达到36%。环境监管水平不断提升。全市114家国省控重点企业全部实现污染源在线监控，实行县、乡、村三级环保网格化管理，环境执法力度不断加大，关停环保违法企业230家，立案查处各类污染环境案件521起。圆满完成APEC会议期间空气质量保障任务。在此，向为改善大气质量付出艰辛努力、做出积极贡献的全市各界，表示衷心的感谢！

民生保障水平不断提高。全年各级财政用于民生的支出达到447.4亿元，占一般公共预算支出的79.4%。就业服务得到加强。积极开展就业困难人员、零就业家庭就业援助，着力做好压减产能涉及职工的安置分流和再就业工作，全年城镇新增就业10万人，农村劳动力转移就业5.2万人，保持高校毕业生登记失业率为零。保障水平不断提高。企业退休人员人均月养老金由1655元提高到1844元；城镇居民基本医疗保险和大病保险最高支付限额达到30万元；提高了城乡低保、农村“五保”供养标准。建立计生特殊家庭“医养扶一体化”服务保障机制。对市内区60周岁以上困难老人和90岁以上高龄老人实施政府购买居家养老服务。开工建设保障房2.7万套，竣工2.48万套，分配入住2.06万套。对267个贫困村实施精准扶贫，5.5万贫困人口稳定脱贫。教育事业均衡发展。学前教育资源不断扩大，新增公办幼儿园学位6750个，学前三年毛入园率达到90%。优质教育资源倍增工程成果显著，12个县（市）区通过国家、省义务教育基本均衡评估验收，二十四中一期、二中二期改造等项目扎实推进。山区教育扶贫工程深入实施，设立家庭经济困难学生生活补贴专项经费，累计发放2061.4万元，资助学生29446人次。中高职教育衔接取得新进展，市职教中心等8所中职学校与省内7所高校实现对接。国家教育信息化公共资源平台试点建设取得阶段性成果。在全省率先颁布实施教育设施规划建设管理条例。文化体育事业日益繁荣。霞光大剧院主体竣工，丝弦剧场投入使用，51个社区文化中心提档升级，文化惠民工程深入实施，《百合岭》、《灯魂》等精品创作成果丰硕；河北美院文化创意产业基地等4家园区入选首批省级示范园区。成功举办第九届省少数民族传统体育运动会，获省运会“五连冠”，石家庄永昌足球队“冲超”成功。医药卫生事业健康发展。全市31家县级公立医院全部实现药品零差率销售；市一院中心院区综合病房楼、市四院新院区、市五院应急救援综合楼等优质卫生资源倍增工程加快推进；国家中医药发展综合改革试验市建设成效显著，创建“国医馆”、“国医堂”做法在全国推广。新农合筹资标准由340元提高到390元，参合率达到98.4%。“单独两孩”政策启动实施。社会治理不断强化。深入实施食品药品安全专项整治，持续开展安全生产隐患排查整改和打非治违行动，各类事故同比下降12%。发挥价格调节基金作用，物价总水平基本稳定。严厉打击各种刑事犯罪，刑事发案率持续下降。实行重大事项社会稳定风险评估制度，有效预防和化解矛盾，保持社会和谐稳定，连续五次入选全国幸福城市前十名，人民群众安全感、幸福感进一步增强。公开承诺的农村饮水安全、缓解“入园难”、助残帮扶等十件实事全面完成。

一年来，我们深入开展党的群众路线教育实践活动，严格执行“八项规定”和“约法三章”。自觉接受人大法律监督、政协民主监督和社会舆论监督，认真办理人大代表建议和政协委员提案，按时办复率和走访率均达到100%。加大廉政建设工作力度，严肃查处“庸懒散拖”行为，效能责任追究359人，发展环境进一步优化。国防动员和双拥共建深入开展，妇女儿童、民族宗教、外事侨务、防震减灾、残疾人、气象、档案、人防等各项事业都取得了新成绩。

各位代表！过去的一年，我市在国内外环境复杂多变、经济下行压力加大、污染治理力度空前的背景下，取得上述成绩实属不易。这是省委、省政府和市委正确领导的结果，是全市广大干部群众共同奋斗的结果。在此，我代表市人民政府，向全市人民，向人大代表、政协委员，向各民主党派、工商联、人民团体和社会各界人士，向驻石解放军、武警官兵和政法干警，向中直、省直机关驻石单位，向关心支持石家庄发展的香港和澳门特别行政区同胞、台湾同胞、海外侨胞、国内外朋友，致以崇高的敬意和衷心的感谢！

回顾2014年工作，虽然大多数经济社会发展指标好于全省平均水平，但仍有地区生产总值、规模以上工业增加值、社会消费品零售总额等指标没有完成预期目标。经济发展长期积累的深层次矛盾和问题尚未根本解决，传统产业拉动能力减弱，新兴产业还没有形成战略支撑，创新发展能力还不够强；县域经济发展不平衡，总体实力偏弱；财政收支矛盾突出，公共服务水平还不够高，城乡居民收入增长还不够快；大气污染依然严重，生态环境治理任务十分艰巨；政府职能转变还不到位，服务意识和作风建设仍需大力加强。对此，我们一定强化问题导向，采取有力措施，认真加以解决，决不辜负全市人民的厚望！

二、2015年主要工作任务

今年是全面深化改革的关键之年，是全面推进依法治国的开局之年，是全面完成“十二五”规划的收官之年。综合分析全年形势，世界经济总体复苏疲弱态势难以明显改观，我国经济运行多重困难和多方面矛盾相互交织，经济下行压力依然较大；我市正处在转型升级、跨越赶超的关键时期，保持经济平稳较快发展的任务十分艰巨。在看到困难的同时，我们更要看到，我国仍处于重要战略机遇期，经济发展进入新常态，总体向好的基本面没有改变；新型工业化、新型城镇化、信息化和农业现代化深入推进，省会行政区划调整顺利完成，为我市经济转型升级开辟了广阔空间；京津冀协同发展进入全面实施阶段，随着交通互联互通、产业协作发展、生态共建共享的深入，必将极大提高省会的创新能力和产业层次；市委确定的中东西三大区域协调发展、“一河两岸三组团”城市布局、工业强市等重大战略深入实施，省会首位度和综合实力不断提升，为率先发展奠定了坚实基础，增添了强大动力。我们一定要顺势而为，乘势而上，保持政治定力，坚定必胜信心，抢抓发展先机，努力开创全市经济社会发展新局面！

政府工作的总体要求是：全面贯彻党的十八届三中四中全会、中央经济工作会议、省委八届九次全会、市委九届六次全会精神，坚持稳中求进工作总基调，以提高经济发展质量和效益为中心，主动适应经济发展新常态，把转方式调结构放在更加重要的位置，紧紧抓住京津冀协同发展的重大机遇，深入实施中东西区域协调发展战略，狠抓改革攻坚，突出创新驱动，推进依法行政，优化两个环境，保障改善民生，维护社会稳定，推动省会工作上水平、创一流，加快转型升级、跨越赶超、建设幸福石家庄步伐，全面完成“十二五”规划目标，为在全省率先全面建成小康社会奠定坚实基础。

今年经济社会发展的主要预期目标是：地区生产总值增长8%，一般公共预算收入可比增长12%，服务业增加值增长10%以上，规模以上工业增加值增长9%左右，固定资产投资增长16%，社会消费品零售总额增长12%，进出口总值增长5%，实际利用外资增长5%。城乡居民人均可支配收入分别增长8%和10%，居民消费价格指数涨幅控制在4%以内，城镇登记失业率控制在4.5%以内，人口出生率控制在14.8％。以内。

提出上述目标，充分考虑了与完成“十二五”规划和率先全面建成小康社会目标相衔接，符合省会发展实际和社会预期，能够更好地适应经济发展新常态。我们一定要全面落实市委九届六次全会部署，坚持把创新发展作为贯穿经济工作的主线，努力在推进工业强市、发展现代服务业、现代农业、壮大县域经济和项目建设上实现新突破。更加注重深化改革开放、释放发展潜力，更加注重项目建设、增强经济发展后劲，更加注重主动融入京津冀协同发展、打造省会发展新优势，更加注重科技成果转化、提高创新驱动能力，更加注重提升公共服务水平、增进民生幸福，努力向全市人民交出一份满意答卷！

围绕实现今年目标，重点做好九个方面工作：

（一）集中实施项目攻坚，强力拉动经济增长。扩大投资规模，优化投资结构，提高投资效益，实现上大项目、上一批大项目的新突破，打造石家庄经济升级版。

狠抓重点项目建设。全年实施亿元以上项目1000个以上，总投资9000亿元，年度完成投资3000亿元。以 “三个一百”重点项目为抓手，实施合力攻坚，确保石药抗肿瘤新药产业化、石煤机二期等100个项目开工建设，格力三期、通用航空产业基地二期、神威医药物流园等100个项目竣工投产，力促石炼化轻烃综合利用一期、河北光纤产业集群等100个前期项目完成各项前期手续。把泸州老窖华北基地、华药青霉素产业升级等单体投资超10亿元的160个产业项目作为重中之重，集中力量保开工、快建设、早投产。筛选一批大项目、好项目列入省重点项目盘子。紧紧抓住国家实施交通建设、清洁能源等“七大工程包”的重大机遇，精心谋划未来三年的支撑性项目，力争市级重点项目储备达到2000项以上。优先保障重点项目建设用地，提高土地利用效率，全年补充耕地3.5万亩。

突出抓好战略性新兴产业。努力“让新芽长成大树”，加快提升新兴产业支撑能力。重点围绕生物医药、高端装备制造、信息技术、新材料、新能源、节能环保等新兴产业，大力推进总投资638亿元的62个项目建设，确保完成年度投资182亿元。抓住建设国家高端生物医药产业集聚发展试点市机遇，制定出台医药产业振兴规划，支持华药、石药、四药、以岭、神威等龙头企业加快发展，确保科仁创新药物产业化等5个项目如期开工，石药制剂扩产改造等3个项目竣工投产，对获得新药证书的企业给予奖励。积极推进中车城轨车辆一期、翼辰铁路配件、欣意电缆等项目建设，确保高端装备制造产业增加值增长18%以上。支持中电科导航、四方通信、旭新光电等骨干企业做大做强，加快发展有源光缆、光电显示玻璃基板、新型连接器开关等高精尖产品，打造中国光纤产业集群。抓好中博、新宇宙新能源汽车项目建设，努力打造中国北方新能源汽车生产基地。推进中广核、河北国威光伏电站建设，力争上海航天机电、山东润峰光伏电站一期工程竣工投用。

提升产业园区承载能力。继续实施开发区产业倍增三年计划，力争高新技术开发区主营业务收入超过2000亿元，经济技术开发区超过1200亿元，循环化工园区超过500亿元，全市开发区主营业务收入增长26%以上。大力提升25个省级以上开发区道路、电力、通讯、供排水等基础设施建设水平，力争高标准实现“九通一平”。积极创建国别型、区域型、产业型特色园区，重点推进栾城浙商机电产业园、藁城翼辰铁料工业园、行唐台湾创新产业园、无极香港皮革制品产业园、元氏中小企业产业园等园区建设。完善园区管理体制和运行机制，提升项目审核、环评、金融等综合服务水平，为企业发展创造良好环境。

（二）深入实施工业强市战略，全力提高质量和效益。坚持走新型工业化道路，改造提升传统产业，发展壮大特色产业，做大做强龙头企业，加快工业经济由规模速度型向质量效率型转变。

推进传统工业改造升级。“让老树开出新花”，提升传统产业装备水平和智慧制造能力。积极引导钢铁、纺织、建材、化工等传统优势产业加大技改投入，推进工业化与信息化深度融合，全年滚动实施千万元以上重点技改项目200项，技改投入达到1600亿元以上。重点抓好高性能纤维复合材料、冀中能源煤制天然气等89个升级改造项目，开发省级新产品、新技术100项以上。深化对标行动，引导各类企业在研发、生产、销售、管理等环节实施全过程对标，争创省级以上标杆指标200项；推进企业管理创新，年内培育5家省级管理创新示范企业。深入实施品牌战略，年内争创中国驰名商标5件、省著名商标80件、省名牌产品70项。支持钢铁、水泥、钙镁行业降耗提质，加快实现转型升级。

做大做强重点骨干企业。对中航通飞、东旭集团、常山生化、欣意电缆等列入省“三个一百”和市“双11”的工业企业，在项目审批、资质认证、招标采购、银企对接等方面提供全方位服务，引导生产要素向企业集聚，加快培育一批国际国内知名品牌。支持石炼化、格力电器等重点骨干企业，积极向总部争取生产计划指标，最大程度实现满负荷生产，充分发挥产能效益，培育一批百亿级、千亿级企业，提高对省会经济发展的贡献率和支撑力。

支持中小微企业加快成长。深入开展中小微企业“服务提升年”活动，积极推广中小微企业小额信用贷款、创业贷款、“保险+信贷”合作等金融服务，完善银担合作机制，全年担保额达到260亿元以上。支持中小微企业创业基地建设，鼓励有条件的县（市）区建设中小微企业创业产业园。实施中小微企业成长培育工程，筛选120家有市场、有潜力的企业予以重点扶持，促其尽快发展壮大。认真落实“个转企、小升规”扶持政策，年内新增规上工业企业160家。全面落实支持民营经济加快发展的政策，充分激发各类创业实体活力，以50家优势企业为重点，支持民营企业向专精新特方向发展，促进民营经济总量做大、质量做优、效益提高。

壮大县域综合经济实力。按照“分类指导、重点倾斜、合力推进”的思路，以做强特色产业集群为重点，以做大产业园区为载体，统筹项目用地、财税金融、环境容量等要素资源，促进各县（市）区扩大经济总量、提高发展质量，力争主营业务收入超50亿元的产业集群达到20个，超百亿元的达到10个以上。支持中部发展现代服务业、高新技术产业、高端装备制造业，打造转型发展示范区；推动东部加快实现工业突破，重点支持晋州纺织、无极皮革、行唐奶业、高邑建陶、新乐食品加工、赵县生物医药、元氏住宅产业化、深泽医药等特色产业扩大规模、集群发展、培树品牌；支持西部提升生态环境质量，完善基础设施，发展绿色工业、生态农业和休闲旅游业，加快矿区石钢新厂区、平山和井陉光伏发电、灵寿纳米节能薄膜、赞皇国家级生态县创建等项目建设，推动中东西三大区域协调发展。

（三）大力发展现代服务业，打造全国现代商贸物流基地。按照服务方式信息化、服务业态高端化、服务内容多样化的思路，提升发展层次，拓宽发展领域，引领现代服务业向价值链高端迈进。

力促现代服务业超常发展。围绕建设国家电子商务示范城市，完善商务云数据中心功能，建设鹿泉数字产业园，抓好汉佳及时惠、财富大厦金融电商、慧聪网等20个电子商务平台建设，力争电子商务交易额达到2800亿元。加快发展跨境电子商务，依托综合保税区，全力申报国家跨境电子商务试点城市。启动国际服务外包经济开发区建设，确保国家软件基地二期扩建项目顺利开工。加快发展现代商贸物流业，突出抓好浙江传化、中储物流中心、京铁亿博物流港等总投资830亿元的75个重点项目；加快国际贸易城建设，力争会展、仓储、国际馆等主要设施建成开业。提升南部综合、正定商贸和西北综合三个省级物流产业聚集区水平，力争年交易额突破500亿元。大力发展金融服务业，鼓励金融机构扩大信贷规模；积极引进国内外金融机构，力争广发银行入驻省会。加强上市辅导服务，确保科林电气、通合电子等10家企业到多层次资本市场上市融资。积极发展健康养老服务业，支持富力健康养生城、南洋生态城、中节能健康城、神威医养综合体等龙头项目建设。加快发展会展业，积极申办通用航空展、京津冀三地电子商务博览会，举办好正博会、药博会等大型展会。

大力提升传统服务业。积极利用新型商业模式改造批发、零售、餐饮等传统服务业，大力发展新兴业态，推进传统服务业跨界创新发展。支持勒泰、万达、万象天成等大型商贸零售企业拓展“线上”业务，开办网上名品店、网上超市、网上地方特色市场。提升南三条、新华集贸、正定小商品等传统批发市场，着力打造集批发零售、展示交易、仓储物流、电子商务于一体的现代化市场。高标准建设商贸流通服务设施，重点抓好总投资1105亿元的21个大型商贸项目，完成年度投资224亿元，支持北人集团加快奥特莱斯、北国商城西扩等项目建设，确保中环广场、高新区中央商务区等项目开工，振西购物中心、阿尔卡迪亚商务广场等建成开业。以大型商务楼宇为依托，引进文化创意、现代商务、广告设计等企业总部，培育一批各具特色的税收“亿元楼”。

努力扩大城乡居民消费。大力推进国家信息消费试点城市建设，围绕养老健康家政、教育文化体育等重点领域，实施智慧社区、数字家庭等六大工程，确保社区光纤网络和行政村宽带实现全覆盖。优化城乡流通网络，在三环周边谋划建设大型超市，新增社区服务网点50家以上；推进电子商务向农村延伸，新建县级配送中心5个、乡村供销超市200家。落实职工带薪休假制度，大力发展休闲旅游、特色旅游，新增星级农家乐50家，旅游业总收入增长15%以上。

（四）加快转变农业发展方式，实现现代农业发展新突破。全面推进国家现代农业示范区建设，大力提升农业产业化、规模化、组织化、品牌化水平，促进农业集约发展、可持续发展。

提升农业综合生产能力。加强基本农田保护，启动永久基本农田划定工作。完成65万亩高标准基本农田建设，大力推广水肥一体化等先进技术，创建50个小麦和48个玉米万亩高产示范片，不断提高粮食生产能力。发展壮大畜牧、蔬菜、林果三大优势产业，挖掘老字号公司品牌价值，支持洛杉奇参与国家生态养殖标准制定，新建双鸽生态养殖园等100个畜禽标准化养殖示范场，完成60家乳粉用标准化奶牛场、30个市级以上现代蔬菜标准园、15个现代果品示范区建设。提高农业科技创新能力，争创国家级农业科技示范园区。大力发展特色农业、节水农业、绿色农业和休闲观光农业，打造2个休闲观光农业示范县、10个都市现代农业园区。加大农产品质量监管力度，新增质量可追溯试点30个。

壮大农业产业化规模。积极把现代产业组织方式引入农业，谋划建设农业产业化项目180个以上，重点抓好以凯隆达为代表的食品加工业、以雨润为代表的农产品物流业，新增市级以上龙头企业20家，农业产业化经营率达到63.1%。全力支持君乐宝上市融资，加快建设三元现代乳品工业园。大力发展各类新型农业经营主体，积极推进适度规模经营，农民合作社达到1.3万家，家庭农场达到1000家。提高农业装备技术水平，发展农业机械专业合作社，主要农作物耕种收机械化水平提高到85.5%。完善农业农村综合信息服务体系，建设信息化示范工程，构建统一的农业网络服务平台。

改善农村生产生活条件。深入开展农村面貌改造提升行动，完成400个省级重点村改造任务。重点抓好正定、西柏坡两个省级片区建设，推进鹿泉、栾城、藁城、矿区四个区村庄全面提升，其它县（市）各打造1个重点片区。实施高铁高速沿线垃圾清理和环境整治专项行动。切实做好历史文化名镇名村的规划保护工作。加强农田水利基本建设，发展节水灌溉面积53万亩。抓好槐河等8条中小河流治理，完成25座小型水库除险加固，治理水土流失面积160平方公里。建设农村公路400公里、户用沼气8400个。加大对271个贫困村的帮扶力度，确保5万人稳定脱贫，平山县脱贫出列。

（五）实施创新驱动战略，加快推进经济转型发展。把创新驱动置于经济发展全局的核心位置，整合创新资源，加快成果转化，贯通产业链条，努力让创新成为驱动发展新引擎。

大力引进创新人才和科技成果。出台实施引进科技创新产业人才的政策措施，为所有创新创业产业人才提供最优惠的激励政策、最优质的金融服务、最优良的创业环境和最优厚的生活待遇。全市新安排6亿元科技成果转化扶持基金，支持入选国家“千人计划”、省“百人计划”的海内外人才到我市创业。促进科技与金融深度融合，探索开展专利权、科技成果入股试点，建立主要由市场决定项目和经费分配机制。以建立完善科技支行、担保机构、保险机构为重点，打造专门支持科技创新的金融服务平台。重点推进以岭院士楼、石药集团等院士工作站、博士后科研工作站建设，支持以岭药业承办好国际络病学大会。

强化企业创新主体地位。支持优势企业开展关键核心技术和重大科技成果联合攻关，推动卫星导航、药用制剂、高端装备制造、光电子等创新型产业集群发展，集中实施重大科技专项25项以上、省级以上科技项目110项以上，力争新增技术创新示范企业和工业设计中心20家。着力抓好技术研究中心和重点实验室建设，年内新培育认定市级以上工程技术研究中心10家以上，争取中电科54所等重点实验室进入国家序列。实施科技型中小企业成长计划，全年培育科技型中小企业2000家以上，选择50家科技型中小企业进行重点培育，力争新认定高新技术企业30家以上，高新技术产业增加值增长14%。

加强科技创新平台建设。充分发挥省会科技大市场“交易、共享、服务、交流”一体化综合服务功能，加强与中国技术交易所等技术转移机构的合作，年内技术合同登记额增长20%以上。各县（市）区都要建设科技型中小企业孵化器，为创新创业人才搭建发展平台、提供优质服务。在高新区、正定新区、鹿泉区等县（市）区建设科技型中小企业产业园，满足孵化毕业企业落地发展需要。厚植创业创新文化，加强知识产权保护，尊重劳动、尊重创造、尊重人才，努力开创大众创业、万众创新的新局面。

（六）加快大省省会建设，推进城镇建设上水平出品位。以建设国家新型城镇化试点城市为契机，大力推进主城出品位、组团提标准、新区见形象、县城求突破，完善城镇体系，加快新型城镇化进程，全市城镇化率达到59%。

拉开城市发展框架。坚持以科学规划为引领、路网建设为先导、互联互通为目标，按照新的区划范围和功能布局，启动产业发展规划、城市总体规划、土地利用规划和生态建设规划修编，划定城市开发边界，优化城市空间布局，形成相互衔接、协调一致的规划体系。本着改革的精神和放权的要求，发挥鹿泉、栾城、藁城三个新区的后发优势，激发内在活力，加快发展步伐。实施南三环辅道贯通、307国道辅线拓宽、南二环东延西拓工程，形成东至藁城、西至井陉和矿区的快速通道；南延体育大街，拓宽改造107国道和308国道，完善主城区连接栾城、正定、新乐的快速路网；实施太行大街北延工程，将主城区、正定新区连为一体，加快城市北跨发展步伐。

完善城市承载功能。高标准规划建设以新客站为中心的城市核心商圈，彰显省会品位和特色。增强主城区道路通达能力，实施和平路高架桥西延和裕华路西延工程，建设槐安路匝道，加快推进新胜利大街建设，改造提升建设大街、仓丰路等市区道路。加快轨道交通建设，确保1、3号线一期工程首开段年底实现“洞通”，完成2号线初步设计评审及报批等前期工作。推进“公交都市”建设，新开辟5条公交线路，启动快速公交建设，建成汇通路公交枢纽站和30个港湾式公交站。提升城市排水管网建设标准，完善胜利大街仓丰路地道桥区域排水设施，对中山路、维明街等路段进行雨污分流改造。谋划建设煤粉高效利用等洁净热源项目，推进热计量入户改造，提升供热保障能力。提高建筑节能标准，推广使用新能源、新技术、新材料。实施市区配电网提升工程，对二环内配电线路进行自动化改造，完善152个老旧小区低压供电设施。实施中山路、太行大街、民心河北线等园林绿化重点工程，在裕华区建设占地208亩的城市公园，在滹沱河南岸建设占地1000亩的生态公园。对滹沱河中华大街至太行大街河道两侧实施绿化美化，打造16公里滨水生态景观带。

提升城市精细化管理水平。全面对标天津，完善城市管理标准体系，提高城市管理规范化、数字化、常态化水平。加快智慧城市建设，加强4G网络推广应用，实施城市百兆光纤工程，扩大“天眼”工程覆盖范围，建设城市管理大数据平台。完善鹿泉、藁城、栾城三个新区数字城管平台建设，尽快实现与主城区“同城化”发展。深入开展房地产市场专项整治，严格规划控制，规范土地市场，确保房地产市场健康有序发展。理顺城管执法体制，推进综合执法，启动市区道路停车管理改革，加大交通秩序、市容环境治理力度。提升城市保洁水平，二环内主次干道机械化清扫保洁率达到80%以上。

加大正定新区建设和古城保护力度。实施城市跨河发展战略，把正定新区作为城市建设的主战场，加快4平方公里先导区建设，尽快集聚产业、集聚人气。统一规划、分步建设规划展馆、科技馆、图书馆、大剧院、青少年活动中心等功能性场馆，确保奥体中心年底投用，国际展览中心、传媒大厦主体封顶，职教园区特教学校竣工、信息技术学校主体完工。加快实施碧水源集团河北总部、宝能中心、河北出版传媒中心等产业项目。进一步完善配套设施，完成北京大街南延，新建续建隆兴大道等12条道路，确保污水处理厂和供水厂建成投用。加大正定古城保护力度，完成南城墙、阳和楼、隆兴寺、开元寺片区改造，启动实施博物馆、历史建筑修缮等9项工程，基本恢复“千年古郡、北方雄镇”的历史风貌。

加快推进县城建设。全面实施“绿化增量、景观提升、精细管理、精品街道打造、基础设施建设”五大攻坚，继续完善县城建设投融资体制，加快城中村和老旧小区改造，每个县建成1座占地150亩以上的综合性公园、3条以上绿化精品街道、1座展示县域风貌的特色建筑，县城生活垃圾无害化处理率达到90%以上，污水处理率达到81%以上。高标准规划建设商务中心、文化中心，所有县城整体上都要实现突破性提升。灵寿、深泽、赞皇力争从全省县城建设重点推进县中出列，赵县、无极争创省级园林县城。稳妥推进乡改镇、村改居工作。落实放宽户口迁移政策，合理引导农业人口向城镇转移，有序推进转移人口市民化。

（七）深化改革开放，增强发展动力和活力。坚持以改革统领全局，以开放活跃全局，努力提高改革开放的深度和广度，打造开放型经济新优势。

继续深化重点领域改革。完善国有资产监管体制，推进经营性国有资产集中统一监管，加快从管资产为主向管资本为主转变，抓好石焦集团搬迁重组、市制酒厂混合所有制改革、北国股份增资扩股和常山股份定向增发工作，完成动力机械厂等5家企业改制重组。深化农村综合改革，积极推进农村土地承包经营权依法有序、适度规模流转，年内土地承包经营权确权登记颁证完成全部工作任务的60%以上。扎实推进供销社综合改革。加快财政体制改革，认真落实新《预算法》，严格实行全口径预算管理；推行县以下机关公务员职务与职级并行制度；扩大政府购买服务范围，积极向出版印刷、咨询评估、检验检测等事务性项目延伸。按要求完成公务用车制度改革。创新投融资机制，整合财政专项资金，通过股权投资基金、政府和社会资本合作（PPP）、第三方担保等融资模式，放大财政资金撬动作用；激活社会资本，支持民间资本发起设立产业投资基金和股权投资基金；搞活县域金融，加快设立服务中小企业的金融机构，积极发展民营银行，筹建晋州农商行，做好深泽、行唐、赵县村镇银行设立工作。继续深化商事制度改革，开展企业准入单一窗口审批试点。稳妥推进资源类民生价格改革，建立居民用水、用气阶梯价格制度。启动不动产统一登记工作。

扎实推进对外开放。实施“走出去”战略，推广苹乐集团境外投资、多元发展经验，支持新大东服装、鼎鑫水泥等建材、纺织服装、原材料生产企业向国外转移，年内新增境外投资企业20家以上。对年进出口额超5000万美元的20家企业实行一对一帮扶，鼓励企业境外商标注册和认证。实施“引进来”战略，主动对接世界500强和国内领军企业，加强与环渤海、长三角、珠三角等重点地区的经济合作。办好全球冀商领袖峰会暨回乡投资洽谈会。年内正定、栾城、藁城、鹿泉、高新区党政主要领导每人至少引进1个10亿元以上项目，其他县（市）区党政主要领导每人至少引进1个5亿元以上的项目。全年引进市外资金增长15%。

主动融入京津冀协同发展。以交通、生态环保、产业三个领域率先突破为重点，推进协同创新，主动承接非首都核心功能疏解，加快融入京津冀城市群步伐。扎实做好京石、津石城际铁路前期工作，加快石济客专建设，力促京石城际铁路早日开工；加密省会高速路网，确保京昆石太高速建成通车、南绕城高速全面开工，启动津石、西阜、平赞和石衡等高速公路建设，完成5条高速公路连接线建设，形成石家庄与北京、天津之间3条以上快速公路通道。强化正定国际机场区域枢纽功能，全力打造联接京津的高铁、高速、空港立体交通体系。深化与京津大气污染联防联控。切实发挥京津冀产学研联盟的桥梁纽带作用，努力形成研发在京津、转化在石家庄的产业链条。加强与北京的产业对接，推进新型中药制剂研究中心、中交财富中心、高邑新材料基地等一批项目在我市落地。深化与中关村的战略合作，积极争取国家集成电路产业投资基金支持，全力推进中关村正定集成电路封装产业基地建设。

高标准建设综合保税区。全部完成2.86平方公里土地征收工作。加快建设综合服务中心和卡口通道、检验检疫区、信息系统等口岸设施，确保海关、检验检疫等机构顺利入驻。按期完成供水、供电、供热等配套基础设施，确保顺利通过国家验收。加大招商引资力度，引进一批科技含量高、经济效益好、符合节能环保要求的外向型企业，重点抓好美国合众速递、颖都皮草等项目建设。依托正定国际机场，发展国际航空服务业，推进“区港一体化”发展，尽快把综合保税区打造成为全省外向度最高、政策最优、功能最全的开放先行区。

（八）强力推进污染治理，实现生态环境明显好转。良好的生态环境是最普惠的民生福祉。必须进一步强化源头严防、过程严管、后果严惩，加快向绿色低碳循环发展新方式转变。

全力改善大气环境质量。继续采取“压煤、抑尘、控车、迁企、减排、增绿”综合措施，科学治霾，精准治污，确保空气质量明显好转。出台大气污染深入治理三年规划，全年削减燃煤270万吨。大力推广新型节能环保锅炉和炉具，加快淘汰10蒸吨及以下燃煤锅炉。严控扬尘污染，市区建筑工地全部实行绿色施工，渣土车辆全部采取全密闭措施，所有露天矿山、采砂场全部实行绿色生产。建立黄标车动态淘汰机制，确保黄标车只减不增。推进主城区工业企业退城进园，加快石钢整体搬迁步伐，确保2017年新厂区建成投产。对钢铁、水泥、电力、玻璃四个行业实行企业排污总量控制，对重点企业的燃煤锅炉、工业窑炉实施除尘、脱硫、脱硝改造，全面执行最新排放标准。

深入实施生态修复工程。加强饮用水源地环境保护，启动岗南、黄壁庄水库上游导污工程，对岗南水库实施全面隔离防护。继续实施河流生态整治工程，重点抓好滹沱河、磁河综合整治，提升汪洋沟、洨河的自净能力，确保河流断面水质全面达标。编制地下水超采综合治理中长期规划，逐步关停自备井，严格控制地下水超采。提升污水处理能力，提高污水排放标准。加快南水北调配套工程建设，建成主城区3座地表水厂和15座县级水厂，主城区春节前用上南水北调引来的长江水。加强山水林田湖综合治理，实施环省会、西部山区、河流绿化美化提升工程，全市完成造林绿化50万亩，森林覆盖率达到37%以上。制定土壤污染防治计划，全面治理农业面源污染。

加强环境保护监督管理。严格落实新《环保法》和《大气污染防治法》，强化环境监管执法力度，开展环境保护大检查，依法严厉打击环境违法行为，切实把污染治理转移到法治轨道上来。加快建设总量减排和大气预报预警两个中心，实行排污总量IC卡控制制度。加强环保网格化属地监管，完善环保监管评价机制，加大污染防治问责力度。执行新的排污费征收标准，推行阶梯差别收费，提高企业主动治污积极性。

（九）着力保障和改善民生，不断增进人民群众福祉。坚持守住底线、突出重点、完善制度，保障基本民生，关注低收入群众生活，保持社会和谐稳定。

完善社会保障体系。努力扩大就业再就业，统筹抓好高校毕业生、农村转移劳动力、城镇困难人员、退役军人等重点人群的就业工作，全年城镇新增就业9.52万人，农村劳动力转移就业5.15万人。开工建设保障性住房2.3万套，基本建成2.2万套，分配入住2万套。推进城镇职工和居民基本医疗保险异地就医即时结算，积极做好工伤保险省级统筹工作。确保城镇职工参加基本养老保险人数达到197.9万人、城镇职工和居民基本医疗保险参保人数达到281万人。扎实推进居家养老、社区养老和集中养老协调发展，新增养老床位3000张，新建标准化居家养老综合服务中心10家。全市农村五保集中供养能力达到70%以上。完善临时救助制度，有效解决群众突发性生活困难。

促进教育公平均衡发展。实施第二期学前三年行动计划，规范居民住宅小区教育设施建设和移交工作。推进优质教育资源提升扩充工程，加快普通中小学现代化学校建设，完成国家、省对11个县（市）区义务教育基本均衡评估认定。深入实施山区教育扶贫工程，重点抓好义务教育与高中段教育、职业教育全面衔接。大力发展现代职业教育，加快市职教园区建设，整合主城区职业教育资源，培养社会急需的技能型人才。推进石家庄学院向应用型技术大学转型，提升重点专业学科服务经济社会发展的水平。扎实推进国家中小学综合质量评价、特殊教育综合改革示范市建设。

提高基本医疗保障水平。深化县级公立医院改革，全面取消药品加成。继续推进优质卫生资源倍增工程，加快市一院正定新区分院、市六院玉村院区等项目建设。实施“国医堂”建设三年行动计划，力争100%的社区卫生服务中心、90%以上乡镇卫生院能够提供中医药服务。提高新农合筹资标准，政府补助资金由每人320元提高到380元，补助资金总额达到21.7亿元，同比增长18.8%，参合率达到95%以上。巩固完善新农合大病保险制度，提高实际报销比例，切实解决因病返贫问题。实施疾病应急救助制度。加强计划生育工作，促进人口长期均衡发展。

加强公共文化服务体系建设。践行社会主义核心价值观，弘扬正能量，引领新风尚。进一步完善基层文化设施，抓好第二批国家级公共文化服务体系示范项目和省级示范县创建工作。加快霞光大剧院建设，年底前投入使用。深入开展“文化进万家”、专业艺术院团下基层演出、公益电影放映等文化惠民活动。加强文艺精品创作，力争更多精品获国家级大奖。加快西柏坡版权产业园等产业园区建设。积极开展全民健身活动，更新健身路径100条。组织好“中超”主场比赛，支持石家庄永昌足球俱乐部健康发展。

积极创新社会治理。加快推进社会诚信、商务诚信和司法诚信建设，建立覆盖全市企业和个人的信用信息体系。扎实开展食品安全示范试点城市和食品药品安全县创建活动，切实保障人民群众饮食用药安全。进一步完善立体治安防控体系，严厉打击违法犯罪行为，不断提升人民群众的安全感。持续开展安全生产专项整治行动，坚决遏制重特大事故发生。推进司法服务进社区，扎实开展“零发案”小区创建活动，建成50个标准化社区。加强市场价格监管，保持物价总水平基本稳定。完善信访绿色通道制度，方便群众表达诉求。大力发展残疾人和慈善事业，年内建设4座慈善爱心超市、400个经常性社会捐助站。积极开展“双拥”创建活动，支持工会、共青团、妇联等人民团体发挥桥梁纽带作用，保障妇幼权益，关心老龄事业，继续做好国防动员、防震减灾、民族宗教、外事侨务、档案、气象、人防、社科、仲裁等各项工作。切实做好“十三五”规划编制工作。

继续办好利民惠民实事。越是困难时期，越要关心群众生活。今年我们将继续集中财力，办好十件惠民实事。实施老旧小区“双解困”工程。解决100个老旧小区3层以上居民用水难问题；解决100个老旧小区无市场化物业管理问题。提高老旧小区供热质量。对全市170个老旧小区的二次管网和换热站进行改造。支持革命老区重点村建设。对175个革命老区重点村基础设施进行改造，提高公共服务水平，解决行路难、饮水难、增收难等问题。保障农村饮水安全。新建农村饮水安全工程376处，解决410个村、50万人的饮水安全问题。提高助残服务水平。在各县（市）及藁城、栾城、鹿泉、矿区新建20个残疾人日间照料中心。增建街旁游园。在中心城区新建10座街旁游园。深化计生特殊困难家庭帮扶。开设就医绿色通道，提供免费健康体检，享受重大疾病住院费用减免和护理补贴。帮扶高校毕业生就业。设立3000万元就业创业专项资金，全年帮扶高校毕业生实现创业2000人以上，提供基层管理岗位800个。开展主城区小学生免费托管试点。在主城区四区各选取5所小学开展免费托管，逐步解决下午放学后小学生无人看管问题。新扩建公办标准化幼儿园。在市内七区、高新区及部分县（市）新扩建50所公办标准化幼儿园，新增学位6000个。

三、全面加强法治政府建设

按照全面推进依法治国新要求，坚决维护宪法法律权威，大力推进依法治市、依法行政，加快建设职能科学、权责法定、执法严明、公开公正、廉洁高效、守法诚信的法治政府。

依法履行政府职能。坚持法定职责必须为，法无授权不可为，上半年市县乡三级政府行政权力清单、责任清单制度实现全覆盖。建立政府法律顾问制度，把合法性审查纳入决策程序。完善政府决策制度，增强政府决策的公开性、透明性和时效性。积极推进综合执法，加大食品药品、安全生产、环境保护、征地拆迁、民间融资等重点领域执法力度，切实维护群众合法权益。坚持依法综合治税，堵塞漏洞，防止逃税，打击骗税，确保应收尽收。对法定职责范围内的行政事务，勇于负责，敢于担当，拿出苦干实干拼命干的劲头抓落实，不出成果不松手，不达目标不罢休。

加大简政放权力度。加快转变政府职能，进一步精简市级行政审批事项，同步落实省取消下放的审批事项，全面取消非行政许可审批和行政监管事项。加快行政审批服务创新，启动市级政府部门行政审批“两集中、两到位”改革，做到目录之外无审批，中心之外无循环，全面提高行政审批效率。加强县（市）区、乡镇政府机关标准化建设，提高行政效能和规范化水平。全面推行“三级平台、两个代办”服务制度，打通服务企业、联系群众“最后一公里”，为企业和群众提供更加高效便捷的服务。

强化行政权力监督。自觉接受市人大的法律监督、工作监督和市政协的民主监督，诚恳接受社会监督和舆论监督。保障和支持审计、监察等部门依法独立行使监督权。对权力集中的部门和岗位实行分事行权、分岗设权、分级授权，强化内部流程控制，防止权力滥用。按照公开是常态、不公开是例外的要求，推进政府信息公开。整合工程建设招投标、土地使用权出让、国有产权交易、政府采购等专业交易市场，建立统一规范的公共资源交易平台。加快电子政务建设，推行企业投资项目网上并联核准制度。正确引导社会舆论，及时回应社会关切，全面提高政府公信力。

加强反腐倡廉建设。加强惩治和预防腐败体系建设，严格落实党风廉政建设“一岗双责”。进一步完善重点部门和关键岗位廉政风险防控措施，强化对人权、事权、财权的制度约束，筑牢法治“篱笆”，防范权力“越线”。坚决纠正“四风”，严格落实 “八项规定”、“约法三章”，严格控制一般性支出，确保“三公”经费只减不增。严厉查处权力寻租、权钱交易和损害群众利益的腐败行为，做到有腐必反、有贪必肃，树立为民务实清廉的良好形象。

各位代表！全面完成2015年任务目标，责任重大，使命光荣。让我们紧密团结在以习近平同志为总书记的党中央周围，在省委、省政府和市委的坚强领导下，紧紧依靠全市人民，奋发有为，开拓进取，加快转型升级、跨越超越、建设幸福石家庄步伐，为在全省率先全面建成小康社会而努力奋斗！
